# Deterministic compressive sampling for high-quality image reconstruction of ultrasound tomography

**DOI:** 10.1186/s12880-017-0206-8

**Published:** 2017-05-25

**Authors:** Tran Quang Huy, Huynh Huu Tue, Ton That Long, Tran Duc-Tan

**Affiliations:** 1 HaNoi Pedagogical University 2, Hanoi, Vietnam; 2 School of Electrical Engineering, VNU International University, HoChiMinh, Vietnam; 3 Faculty of Electronics & Telecommunications, VNU University of Engineering & Technology, Hanoi, Vietnam

**Keywords:** Mammography, Ultrasound tomography, Inverse scattering, Distorted born iterative method (DBIM), Deterministic compressive sampling (DCS)

## Abstract

**Background:**

A well-known diagnostic imaging modality, termed ultrasound tomography, was quickly developed for the detection of very small tumors whose sizes are smaller than the wavelength of the incident pressure wave without ionizing radiation, compared to the current gold-standard X-ray mammography. Based on inverse scattering technique, ultrasound tomography uses some material properties such as sound contrast or attenuation to detect small targets. The Distorted Born Iterative Method (DBIM) based on first-order Born approximation is an efficient diffraction tomography approach. One of the challenges for a high quality reconstruction is to obtain many measurements from the number of transmitters and receivers. Given the fact that biomedical images are often sparse, the compressed sensing (CS) technique could be therefore effectively applied to ultrasound tomography by reducing the number of transmitters and receivers, while maintaining a high quality of image reconstruction.

**Methods:**

There are currently several work on CS that dispose randomly distributed locations for the measurement system. However, this random configuration is relatively difficult to implement in practice. Instead of it, we should adopt a methodology that helps determine the locations of measurement devices in a deterministic way. For this, we develop the novel DCS-DBIM algorithm that is highly applicable in practice. Inspired of the exploitation of the deterministic compressed sensing technique (DCS) introduced by the authors few years ago with the image reconstruction process implemented using *l*_1_ regularization.

**Results:**

Simulation results of the proposed approach have demonstrated its high performance, with the normalized error approximately 90% reduced, compared to the conventional approach, this new approach can save half of number of measurements and only uses two iterations. Universal image quality index is also evaluated in order to prove the efficiency of the proposed approach.

**Conclusions:**

Numerical simulation results indicate that CS and DCS techniques offer equivalent image reconstruction quality with simpler practical implementation. It would be a very promising approach in practical applications of modern biomedical imaging technology.

## Background

Since Wilhelm Roentgen discovered the X-ray beam in 1885, there has been a big leap in the clinical diagnostic field in which more advanced technologies for Biomedical imaging applications have been developed, e.g. Magnetic Resonance Imaging (MRI), Computed Tomography (CT), Ultrasound Tomography (UT), Electron Paramagnetic Resonance (EPR), etc.… Among the newly developed techniques, ultrasound imaging techniques have become the widely used tool in the health sector due to its implementation ability for diagnostic and therapeutic as well as its many advantages such as low cost, non-invasive nature, painless test, mobility and fast diagnosis.

Ultrasound imaging which uses sound waves in the range between 20 kHz and 1 GHz is commonly used since the development of sonar in 1910. Based on the principle of sonar, one of the techniques that can be widely used is B-mode imaging [1]. This technique is used for non-destruction evaluation and biomedical imaging. B-mode image represents a qualitative change of acoustic impedance function. As a result of this change, it allows to distinguish between environments in the region of interest. However, this imaging technique, using feedback of sound waves when encountering target, only provides the qualitative information of the imaged targets. Meanwhile, ultrasound tomography, based on inverse scattering technique, provides the quantitative information of those targets. Indeed, when sound waves encounter a heterogeneous environment, some of the energy will then be scattered in all directions. The scattered data will be obtained by the receivers which are set up around the target of interest. Therefore, a set of measurements of the scattered field is obtained. Inverse scattering problem includes estimating the distribution of acoustical parameters (e.g. speed of sound, attenuation and density) to reconstruct the target of interest in the inhomogeneous environment. This technique allows a more detailed description of the imaged target. Instead of using acoustical impedance parameter in B-mode imaging, it uses one of parameters of acoustical properties. Therefore, acoustic tomograms display quantitative information of the target under examination.

Although ultrasound tomography has many advantages, this technique has not been widely applied in practice. One of the reasons is the lack of applications that can take advantage of inverse scattering techniques. Currently, the main application of this technique is only for breast imaging in women to detect cancer-causing cells [2–4]. Another limitation of inverse scattering techniques is the lack of efficient and powerful calculation methods. Inverse scattering techniques have high computational complexity and it is also the main reason that there is only a certain number of commercialized tomography devices. Hence, state-of-the-art inverse scattering techniques primarily focus on reducing the computational complexity and constantly improving the quality of imaging. Most of research work on ultrasound tomography are based on Born approximation. Born Iterative Method (BIM) and Distorted Born Iterative Method (DBIM) are well-known for diffraction tomography [5]. The DBIM is a quantitative approach in image reconstruction of the very small target. In this method, the background medium is considered inhomogeneous and is updated with each iteration. Therefore, the equation for Green’s function and the equation for incident field are updated with each iteration.

Compressed sensing (CS), which is introduced by Candes and Tao [6] and Donoho [7] in 2006, could acquire and reconstruct sparse signals at a rate lower than that of Nyquist. Random measurement approach in the detection geometry configuration is proposed in [8, 9]. A set of measurements of the scattered field is performed using sets of receiver’s random positions. This method can reduce the computational complexity and improve the quality of the reconstruction of the sound contrast, compared to the linear measurement method. However, this method does not well de-noise and the implementation of compressed sampling technique based on random sampling leads to a difficult restriction on the hardware of the ultrasound tomography system. It means that the probes will have to be randomly distributed on the measurement system. This problem is difficult to implement because in order to create a random sequence, one must use a hardware random number generator (HRNG) or a pseudo-random number generator (PRNG). The implementations of HRNG and PRNG in practice are very complex. In [10], the authors proposed to use a deterministic measurement matrix, which is deterministic, instead of random one. The elements of this deterministic measurement matrix are chosen from the sequence generated by a logistic deterministic system. Previous simulated results indicate that this approach offers a very good performance compared to the random method. Moreover, using deterministic CS system inherits a simpler hardware implementation than using the random one. The hardware implementation of the deterministic generator is just a simple nonlinear circuit which is much simpler than a random generator. In this paper, based on the compressed sensing technique, we propose a methodology which helps to determine the locations of transmitters and receivers in a deterministic way for ultrasound tomography. Then, we develop a novel DCS-DBIM (Deterministic CS-DBIM) algorithm that is highly applicable in practice. As a result, this approach offers a very high performance, compared to the conventional DBIM method.

## Methods

### Distorted born iterative method

#### Proposal of measurement configuration

**Fig. 1:**
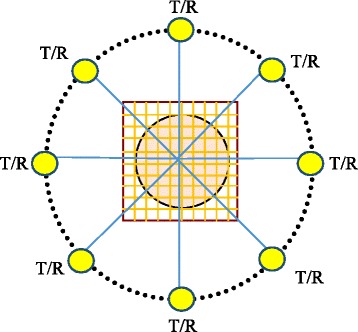
Geometrical and acoustical configuration

Consider the setting of transmitters and receivers in Fig. 1.

As shown in Fig. 1, the measurement configuration is arranged circularly around the target. The pressure signal from a transmitter is propagated, scattered and measured by receivers. In principle, transmitters and receivers can be distributed uniformly, randomly, or deterministically based on the measurement configuration. Besides, the number of transmitters and receivers depends on different scenarios, in line with practical requirements. It should be noticed that the number of transmitters and receivers should not be so large because the measurement system will be complex. In addition, a large amount of computation and memory requirement are needed to store and process the data. It should be also noticed that the locations of transmitters and receivers may be either different or identical. However, with current ultrasound transducer technology, one transducer can both transmit and receive ultrasound signals. In other words, the location of transmitter and receiver can be identical.

For the proposed measurement configuration, we assume that there are M transmitters and N receivers. M transmitters are arranged around the target to obtain complete information about the target at different angles. Transmitter-receiver procedure of ultrasound wave is performed as follows. Initially, only the first transmitter transmits ultrasound signal and all receivers (N) will simultaneously collect the scattered ultrasonic signal. As a result, we get the 1st set of measurements (i.e. N measurements) at the first position of the transmitter. The second transmitter then begins operating and all receivers simultaneously obtain scattering signal at the second position of the transmitter. At the end of this procedure, we obtain the 2nd set of measurements (i.e. 2 × N measurements). The process continues until the last transmitter (i.e. M^*th*^) where we will obtain M sets of measurements (i.e. M × N measurements). With these measured values, full information about the target at different angles around the target are received.

The measured data will then be brought to DBIM to estimate the sound contrast. The change of the sound speed would be utilized to detect any tissue if exists. In this paper, the transmitters and receivers are working under the condition of homogeneous background medium, i.e. water, where there is a target (strange tumor). This target has sound contrast different from the background (denoted as ∆*c*) which is embedded in this medium. Because DBIM is used to detect strange tumors in early stage in this paper, sound constrast compared to the background medium is usually very small. Therefore, in our scenarios, which will be discussed in section Results below, value of ∆*c* will be selected to a few percents.

### DBIM method

Because of the well-known properties, Bessel function [11] is usually used in the numerical simulation as a transmitted signal called Incident Wave whose frequency is *f*. Wavelength λ of this wave is calculated by

$$ \lambda ={c}_0/ f, $$

where *c*_0_ is the sound speed in background medium. For homogeneous medium, the received signal at the receiver is the Incident Wave itself. In presence of tumors, the medium becomes inhomogeneous; when the incident wave hits the target, the following two situations may occur: if the target size is much larger than the wavelength of the incident wave, it is reflected; if the target size is less than or equal to the wavelength of the incident wave, it is scattered in all directions around the target. Notice that ultrasound frequency for clinical diagnosis is in the range from 20 KHz to 12 MHz. As a result, the wavelength ranges from 6.2 μm to 74.2 mm if sound speed in the background is 1484 m/s. Born iterative method is used to find out the linear relationship of the scattered pressure difference and the sound contrast difference. The key of this method is that the scattering signal is considered to be very small in comparison with the incident signal. This is entirely consistent with the real requirements which need to detect strange tumors in early stage. Therefore, we consider to reconstruct targets with very small sound contrast, i.e. scattering signal is very small.

The wave equation of the scheme discussed above can be expressed by

1 $$ \mathrm{p}\left(\overrightarrow{r}\right)={\mathrm{p}}^{\mathrm{inc}}\left(\overrightarrow{r}\right)+{\mathrm{p}}^{\mathrm{sc}}\left(\overrightarrow{r}\right), $$

where ${\mathrm{p}}^{\mathrm{sc}}\left(\overrightarrow{r}\right)$, ${\mathrm{p}}^{\mathrm{inc}}\left(\overrightarrow{r}\right)$, and $\mathrm{p}\left(\overrightarrow{r}\right)$ are the scattered, incident, and total signals respectively. It can be seen that the known data arethe total signal and the incident signal. However, what we concern about is the reconstruction of the unknown target T (r) from the obtained data. This is the inverse problem.

Consider the wave numbers of the background and target mediums to be *k*_0_ and *k* (*r*) respectively. According to [12], inhomogeneous differential equation has the form

$$ \left({\mathit{\nabla}}^2+{k}_0^2(r)\right) p(r)=- O(r) p(r) $$

Green function is an effective method to solve inhomogeneous differential equation. Therefore, it is used to find out the nonlinear relationship of the scattered signal and the target based on the total and incident signals. Eq. (1) can be rewritten in details using the Green function G_0_ (·)

2 $$ \mathrm{p}\left(\overrightarrow{\mathrm{r}}\right)={\mathrm{p}}^{\mathrm{inc}}\left(\overrightarrow{r}\right)+{\displaystyle \iint}\mathrm{T}\left(\overrightarrow{\mathrm{r}}\right)\mathrm{p}\left(\overrightarrow{{\mathrm{r}}^{\hbox{'}}}\right){\mathrm{G}}_0\left({k}_0,\left|\overrightarrow{r}-\overrightarrow{{\mathrm{r}}^{\hbox{'}}}\right|\right)\mathrm{d}\overrightarrow{{\mathrm{r}}^{\hbox{'}}} $$

Notice that when the background medium is homogenerous, G_0_ is the 0-th Hankel function of the first kind and described by

3 $$ \begin{array}{l}{\mathrm{G}}_0\left({k}_0,\left|\overrightarrow{\mathrm{r}}-\overrightarrow{{\mathrm{r}}^{\hbox{'}}}\right|\right)=\frac{- i}{4}{H}_0^{(1)}\left({k}_0\left|\overrightarrow{\mathrm{r}}-\overrightarrow{{\mathrm{r}}^{\hbox{'}}}\right|\right)=\\ {}\frac{- i}{4}\sqrt{\frac{2}{\pi {k}_0\left|\overrightarrow{\mathrm{r}}-\overrightarrow{{\mathrm{r}}^{\hbox{'}}}\right|}}{e}^{i\left({k}_0\left|\overrightarrow{\mathrm{r}}-\overrightarrow{{\mathrm{r}}^{\hbox{'}}}\right|-\pi /4\right)}.\end{array} $$

T (r) in Eq. (2) is the target function that needs to be estimated. It can be calculated as follows:

4 $$ \mathrm{T}\left(\mathrm{r}\right)=\left\{\begin{array}{l} k{(r)}^2\hbox{--} {k}_0^2={\omega}^2\left(\frac{1}{c^2}-\frac{1}{c_0^2}\right)\kern0.5em  if\  r\le R\\ {}0\kern6.36em  if\  r> R\ \end{array}\right. $$

Equation (4) indicates that the ideal target function depends on the frequency of the incident signal (ω = 2π*f*) and the sound speed difference of the background medium (*c*_*0*_) as well as the target medium (*c*). In order to calculate in details each pixel inside the region of interest, scattering wave equation (2) needs to be discreterized. The method of moment (MoM) which uses basic sinc function is used to solve this problem.

The total pressure field in the observed mesh area (*N* × *N* points) can be expressed by

5 $$ \overline{p} = \left(\overline{I}-\overline{C}. D\left(\overline{T}\right)\right){p}^{inc}, $$

where $\overline{C}$ is the Green matrix showing the interactions among pixels, *Ī* is unit matrix, and *D* (·) returns a square diagonal matrix of the input vector. The scattered signal in the form of *N*_*t*_ *N*_*r*_ × *1* vector is described by

6 $$ {\overline{p}}^{sc}=\overline{B}. D\left(\overline{T}\right).\overline{p}, $$

where $\overline{B}$ is the Green matrix showing the interaction of all pixels to the receiver. We have to determine two parameters $\overline{p}$ and $\overline{T}$ in Eqs. (5) and (6).

By rewritting these equations, we have [13]

7 $$ \varDelta {p}^{sc}=\overline{B}. D\left(\overline{p}\right).\varDelta \overline{T}=\overline{M}.\varDelta \overline{T}, $$

where $\overline{M}=\overline{B}\cdot D\left(\overline{p}\right)$. For a transmitter and a receiver, we formulate a matrix $\overline{M}$ and a scalar value *Δp* ^*sc*^. The target function $\overline{T}$ has *N* ^2^ variables corresponding to the number of pixels in the region of interest. It can be estimated by:

8 $$ {\overline{T}}^n={\overline{T}}^{\left( n-1\right)}+\varDelta {\overline{T}}^{\left( n-1\right)}, $$

where *n* and *n*-1 are two consecutive discrete-time points. $\varDelta \overline{T}$ is estimated by using Tikhonov’s regularization [14]:

9 $$ \varDelta \overline{T}= \arg \underset{\Delta \overline{T}}{ \min }{{\left\Vert \Delta \overline{p}\right.}^{sc}}_t-\overline{M_t}\Delta \overline{T}\left\Vert {}_2^2\right.+\gamma {\left\Vert \Delta \overline{T}\right\Vert}_2^2, $$

where $\varDelta {\overline{p}}^{sc}$ is the difference between estimated and measured scattered signals whose size is (*N*_*t*_ *N*_*r*_ × 1). Besides, measurement results are assembled in a matrix form ${\overline{M}}_t$ of (*N*_*t*_ *N*_*r*_ × *N* ^2^) elements and γ is the regularization factor that needs to be carefully chosen.

The DBIM procedure is presented in **Algorithm 1**.
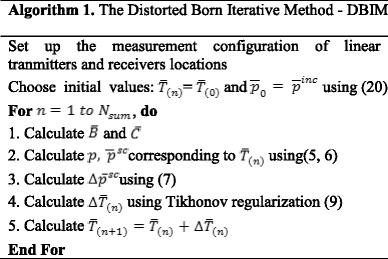


### Deterministic compressive sampling

#### Fundamentals of compressive sampling

Compressive Sampling (CS) or known as Compressed Sensing [15] allows for exactly recovering signal *v* ∈ ℝ^n^ from a small number of “random measurements” *u* ∈ ℝ^m^ (m < < n), which are defined as follows:

10 $$ u = \Phi v, $$

where Φ is a m × n matrix called sampling basis. The columns of Φ have entries equal to 1 at random positions; the entries at other positions are null. This structure means that measurements are done randomly.

The core problem of compressive sampling is that assuming *v* has sparse representation in an orthonormal basis Ψ, i.e.

11 $$ v = \Psi w, $$

in which, *w*, also known as sparse, only has s < m ≪ n non-zero coefficients. Note that s is the sparsity degree of the signal *u* and the number m of required measurements is normally higher than s. Compressive sampling theory shows that this sparse property allows accurate recovery *w* with overwhelming probability success [16]. In particular, sensing basis must have incoherent property to the model basis Ψ [17]. This property is guaranteed by the randomness of the non-zero components in Φ. Therefore, the problem can be written as follows:

12 $$ u = \Phi \Psi w= Aw, $$

where *A* ∈ *R* ^mxn^ is full-rank, i.e. the m rows of *A* are linearly independent. By these settings, Eq. (12) is solved for *w* with *w*-sparse constraint. Once *w* is obtained, *v* can be calculated from (11). With *A* satisfying “Restricted Isometry Property (RIP)” condition which is first established by Candes et al. [16], the CS problem can be solved through *l*_0_-minimization technique:

13 $$ \widehat{w}= \arg \underset{w\in {R}^n}{ \min}\left\Vert w\left\Vert {}_{l_0}\right.\right.\mathrm{subject}\ \mathrm{t}\mathrm{o}\  u= Aw, $$

where *l*_0_ norm is defined as ${\left\Vert w\right\Vert}_{l_0}:={ \max}_i\left|{w}_i\right|$. In this paper, instead of solving (13), the sparsest solution $\widehat{v}$ of (12) can be found by solving the *l*_1_-minimization problem [18]:

14 $$ \widehat{w}= \arg \underset{w\in {R}^n}{ \min}\left\Vert w\right.\left\Vert {}_{l_1}\right.\;\mathrm{subject}\ \mathrm{t}\mathrm{o}\  u= Aw, $$

in which, the *l*_1_ norm is defined as $\left\Vert w\right\Vert {l}_1:={\displaystyle {\sum}_{i=1}^n\left|{w}_i\right|.}$

Equation (12) is introduced under the assumption that the exact form of the reconstructed signal is given. This is rarely the case in practice, because the measurements are often affected by noise. To reconstruct the signal in case of noisy measurements, we have:

15 $$ u = Aw+ e, $$

where *e* represents the noise ${\left\Vert e\right\Vert}_{l_2}\le \varepsilon$. *l*_1_ problem in presence of noise can be expressed as follows [18]:

16 $$ \widehat{w}= \arg { \min}_{w\in {R}^n}{\left\Vert w\right\Vert}_{l_1}\mathrm{subject}\ \mathrm{t}\mathrm{o}\; u={\left\Vert u- Aw\right.}_{l_2}\le \varepsilon $$

### Compressive sampling using deterministic filters

In this paper, we use a deterministic basis generated by a pseudo random sequence instead of pure random basis. The advantage of this method in comparison with CS is that it offers a simpler hardware design, because of the deterministic nature of the proposed measurement system. In fact, to facilitate the practical implementation, researchers focused on the measurement system design whose properties are not completely random or completely deterministic. The first work exploited this view is introduced in [11], followed by others [19, 20]. In the work [21], the authors offer some simple criteria to design a deterministic compressed sampling system that ensures the ability to successfully reconstruct sparse signals. Overall, the deterministic compressed sampling technique inherits some advantages over random one, such as more effective recovery time, clear structure, efficient storage and tighter recovery limit [20].

Because of simpler hardware implementation of a deterministic system compared to a random one’s, in this work, the matrix Φ is constructed using the deterministic method. To perform this, we consider a dynamic deterministic system with deterministic nonlinear characteristic; as its dynamic is very sensitive to initial conditions, its output has random-like property [22]. Note that the reconstruction accuracy of deterministic compressed sampling technique is ensured like the random one [23]. To construct Φ, we consider a Logistic map based dynamic structure that generates a very simple deterministic sequence which is transformed into a sequence that would have a Gaussian-like behavior [22, 23]. The following Logistic map sequence is used as follows

17 $$ q\left( n+1\right) = \rho q(n)\left(1- q(n)\right), $$

We then convert it by the Logit Transform to become Gaussian-like as

18 $$ {q}_G(n)= \ln \left[\frac{q(n)}{1- q(n)}\right] $$

***Remarks***: When the control parameter ρ in (17) equals to 4, the dynamics described by (17) is deterministic; this means that the dynamic of (17) is very sensitive in regard of the initial condition *q* (0). A very small change in *q* (0) would largely change *q* (*n*) in a relatively short time. More detailed construction of Φ from *q*_*G*_ (*n*) can be found in [22]. Finally, the image reconstruction is performed using sparse approximation algorithms.

### The deterministic compressive sampling DBIM

#### Measurement configuration

Assume that there are *N*_*t*_ × *N*_*r*_ transducers where *N*_*t*_ is the number of transmitters and *N*_*r*_ is the number of receivers (that gives *N*_*t*_ × *N*_*r*_ measurements). We need to reconstruct the target which is divided into *N* pixels vertically and horizontally, i.e. *N* ^2^ variables. There are three cases for the arrangement of transducers and transmitters that are required for consideration.

Firstly, if *N*_*t*_ × *N*_*r*_ is larger than *N* ^2^, the number of equations is greater than the number of variables. This is the over-determined problem and it is called *a dense scattering domain*. In this configuration, the amount of scattered information obtained around the target is large, from which we can calculate and easily reconstruct the target image. However, a large number of transmitters and receivers is required. This high number of devices makes the imaging system much more complex in terms of setting up the measurement equipment and of large required processing time consuming in target image reconstruction. Therefore, this configuration is less likely used in practice.

Secondly, if *N*_*t*_ × *N*_*r*_ is approximately or equals to *N* ^2^, the number of equations equals to the number of variables. This case is called *a moderate scattering domain*. For the high-resolution images in this paper, i.e. large *N* ^2^, we still need in this situation a relatively large amount of transducers to give enough measurement for correctly reconstructing images.

Thirdly, if *N*_*t*_ × *N*_*r*_ is smaller than *N* ^2^, i.e. the number of equations is smaller than the number of variables, this is the under-determined problem. When *N*_*t*_ × *N*_*r*_ is significantly smaller than *N* ^2^, we call this *a sparse scattering domain*, that is, transducers is sparse on the measurement system due to the small number of transducers and the amount of scattered information is less and sparser than the case of the *dense and moderate scattering domains*. Define the compressed ratio *r* as the ratio of *N*_*t*_ × *N*_*r*_ and *N* ^2^, i.e.

19 $$ r = \frac{N_t{N}_r}{N^2}. $$

It is clear that for the *sparse scattering* problem, *r* < 1. When *r* is small, the measurement system is simple and the collected data is of small size. Consequently, the system set-up and calculation are less complex. Henceforth, we need to focus on the case of the smallest value of *r*, with which, the imaging system is the simplest and it ensures the acceptable image reconstruction.

**Fig. 2:**
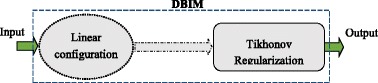
The implementation process of the conventional method

**Fig. 3:**
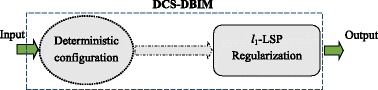
The implementation process of the proposed method

The implementation processes of the conventional method and the proposed method are shown in Fig. 2 and Fig. 3 respectively. In both figures, the Input represents the ideal target function and the Output represents the reconstructed target function. Nevertheless, in Fig. 2, the ideal target function of interest is reconstructed using Tikhonov regularizations while it is reconstructed using sparse-based approximation techniques such as *l*_1_ optimization or greedy Algorithms in Fig. 3. Besides, the measurement configuration of linear transmitter-and-detector locations is used in conventional method and the measurement configuration of transmitters and detectors with linear transmitter locations and deterministic detector locations is used in the proposed method.

The signal accurate reconstruction of the CS is guaranteed if the *sparse domain* is not associated with the sampling domain [6, 7]. In [24], the authors compared the efficiency of sparse signal recovery methods, namely *l*_1_-LSP, MOSEK, PDCO-CHOL, PDCO-LSQR, *l*_1_-MAGIC and HOMOTOPY, and MOSEK. Their results show that *l*_1_-LSP method outperforms others by its fast reconstruction and low computational complexity. Moreover, this also works effectively in large dense problems. With these observations, the DBIM method can be employed for not only detecting small scale targets (like breast cancer) but also for other larger-scale applications. Therefore, in this paper, we use *l*_1_-regularized least-squares programs (*l*_1_-LSP) [24] for reconstructing the image target.

#### l_1_-regularized least-squares and DCS-DBIM procedure

The *l*_1_-LSP solves *l*_1_-regularized least squares problems using the truncated Newton interior-point method and has the form as follows:

20 $$ min\left\Vert Aw- u\left\Vert {}^2\right.\right.+\zeta {\left\Vert w\right\Vert}_1, $$

where *A* is a data matrix in dense or sparse format with n columns and m rows; *w* is a vector of length n; and *u* is a vector of length m. In the DCS-DBIM approach, *A*, *w*, *u* are ${\overline{M}}_t$, $\varDelta \overline{T}$, and $\varDelta {\overline{p}}_t^{sc}$ respectively. It is noted that the size of ${\overline{M}}_t$ is dependent on the number of transmitters (*N*_*t*_), receivers (*N*_*r*_) and variables (*N* ^2^). In other words, it is changed in different scenarios. In the DCS-DBIM form, *l*_1_ problem is expressed as

21 $$ \varDelta \overline{T}= \arg \underset{\Delta \overline{T}}{ \min }{{\left\Vert \Delta \overline{p}\right.}^{sc}}_t-\overline{M_t}\Delta \overline{T}\left\Vert {}_2^2\right.+\zeta {\left\Vert \Delta \overline{T}\right\Vert}_1, $$

where ζ is the regularization parameter in *l*_1_ problem. The selection of regularization parameter ζ is crucial because it has a great influence on the image reconstruction quality. The large value of ζ will make the reconstructed image rough and the small value of ζ will result in high computational complexity. It is noted that the inverse solver matrix ${\overline{M}}_t$ is changed at each iteration. In fact, in DBIM, the Green function is updated in each iteration; therefore, matrices *B* and *C* are changed, leading to the variation of ${\overline{M}}_t$ at each iteration.

In this context, the regularization parameter ζ also changes at each iteration. It is chosen as a function of the forward error. For the computation process, the Rayleigh quotient is recursively iterated; the value σ_0_ of the first singular value of the inverse solver matrix $\overline{M_t}$ is estimated; ζ is then selected as given in [25]. In the simulation scenario presented in this paper, the value of ζ for the first iteration is chosen as 10^−15^. The DCS-DBIM procedure is presented in **Algorithm 2**.
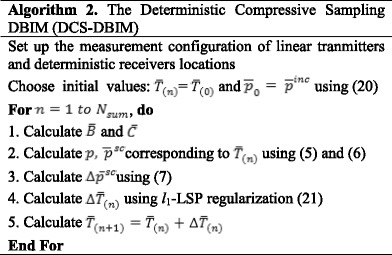


## Results

### Parameters set up

The frequency of the incident signal, *f* = 1 MHz, is selected as in a previous experimental work [26]. It is known that the convergence rate of DCS-DBIM method depends on the tolerated reconstruction error. For large error, the convergence is fast; it slows down when the error is fixed smaller. In any case, after a few iterations, the normalized error reaches a floor. Reducing this floor value requires the reduction in the tolerated distortion of reconstructed images in the *l*_1_ algorithm. Furthermore, it will lead to a more complex computational procedure and as consequence, a much longer imaging time.

The propagation speed of ultrasound wave in the women breast environment is actually in the range of 1350 m/s to 1600 m/s (in the background medium of about 1484 m/s) [27], that is, the difference in the propagation speed in women breast is in the range from 0 to 15.6%. Therefore, choosing the wave propagation speed difference of 5% is reasonable.

The main limitation of the DBIM approach is that the divergence issue occurs when ∆φ > π, where $\varDelta \varphi =2\omega \left(\frac{1}{c}-\frac{1}{c_0}\right) R$ [28]. Therefore, the incident frequency must satisfy $f<\frac{c_0}{2 d \times \%\varDelta c}$. The incident pressure for a Bessel beam of zero order in two-dimensional case is described by

22 $$ {\overline{p}}^{inc} = {J}_0\left({k}_0\left| r-{r}_k\right|\right), $$

where *J*_0_ is the 0th order Bessel function and |*r* − *r*_*k*_| is the distance between the transmitter and the *k* ^th^ point in the range of interest.

Based on the above discussions, for the simulations, the following parameters are finally chosen: frequency *f* = 1 MHz; the total number of iterations *N*_*sum*_ = 8; N = 21 (i.e. Number of variables is *N* ^2^ = 21 × 21 = 441); Target diameter = 7.3 mm; Sound contrast 5%; Signal-to-noise ratio SNR = 20 dB; Distances from transmitters and receivers to the center of the target are 100 mm. The numerical simulation program used is MATLAB running on a PC with Intel core i3 processor and 2 GB RAM.

### Performance evaluation of the DCS-DBIM and DBIM

**Fig. 4:**
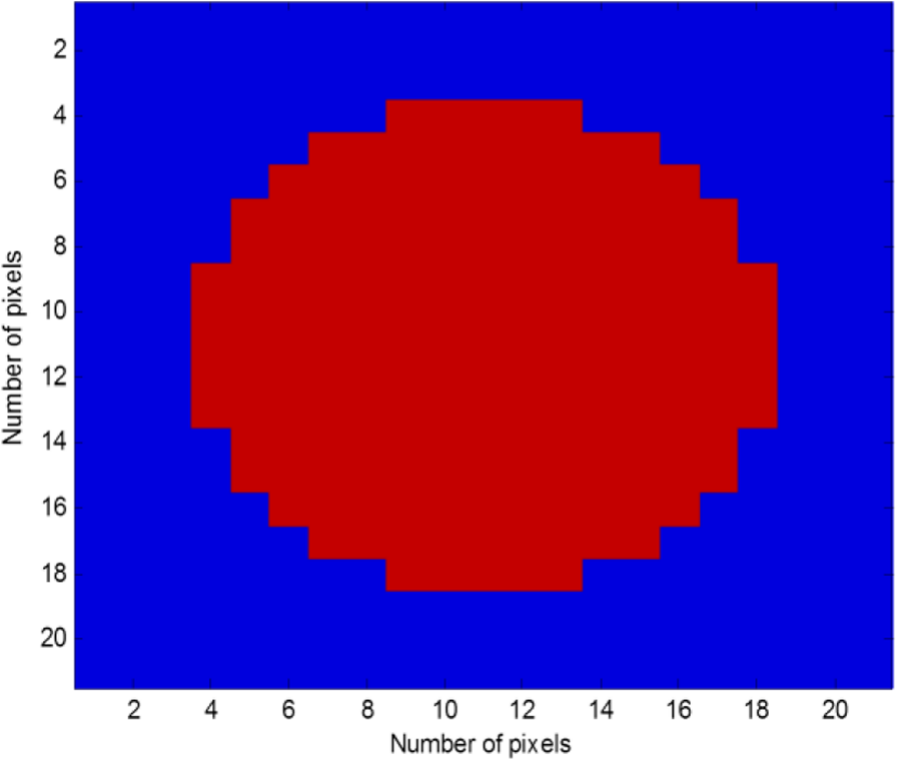
Ideal target function (*N* = 21)

**Fig. 5:**
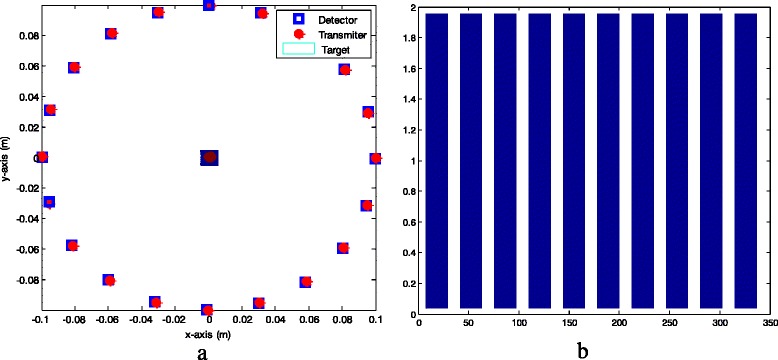
**a** Conventional configuration of transmitters and detectors using linear transmitter-and-detector locations (*N*_*t*_ = *N*_*r*_ = 20, *r* = 0.826); **b** Histogram of linear detector locations over full circle (*N*_*r*_ = 20)

The ideal target function T (*r*) described in Eq. (4) is shown in Fig. 4 where it is placed at the center of the meshing area with *X* and *Y* indicating the pixels coordinates. The conventional linear configuration of transmitters and for the case of *N*_*t*_ = *N*_*r*_ = 22 is presented in Fig. 5a while histogram of linear detector locations over full circle in case of *N*_*r*_ = 22 is shown in Fig. 5b.

**Fig. 6:**
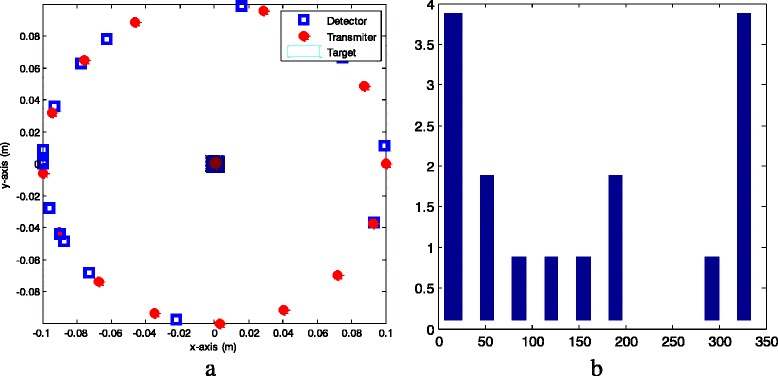
**a** Proposed configuration of transmitters and detectors using linear transmitter locations and detector DCS locations (*N*_*t*_ = *N*_*r*_ = 16, *r* = 0.581); **b** Histogram of detector DCS locations over full circle (*N*_*r*_ = 16)

We propose a deterministic under-sampling configuration of detectors, in which the number of detectors is smaller than the number of detectors in the conventional configuration in Fig. 6. With a reduced number M of measurements and the less computational complexity in the iteration process, the proposed configuration sustains a quality of the reconstruction comparable to that obtained by the conventional configuration. Notice that the transmitters are still placed at equal distance as in the conventional configuration. The proposed configuration of transmitters and detectors with linear transmitter locations and detector DCS locations in case of *N*_*t*_ = *N*_*r*_ = 16 is depicted in Fig. 6a. Meanwhile, Fig. 6b shows the histogram of detector DCS locations over full circle in case of *N*_*r*_ = 16.

To quantify the efficiency of the proposed approach, we acquire the target functions to obtain experimental data to be used in the iterative reconstruction of target image. Then, the error in the reconstructed image is determined and compared to the original image at each iteration. Suppose that *m* is a *P* × *Q* original image (i.e. ideal target function) and $\widehat{m}$ is the reconstructed image. The normalized absolute error can be defined as:

23 $$ \varepsilon =\frac{1}{ P xQ}{\displaystyle \sum_{i=1}^P}{\displaystyle \sum_{j=1}^Q}\frac{\left|{m}_{i j}-{\widehat{m}}_{i j}\right|}{\left|{m}_{i j}\right|} $$

The normalized absolute errors and runtimes of the DBIM and DCS-DBIM methods through iterations with different number of transmitters (*N*_*t*_) and receivers (*N*_*r*_) are presented in Table 1. These simulation results have shown that after *N*_*sum*_ iterations, in some cases, the runtime of the DCS-DBIM method is significantly larger than that of the DBIM method. The price to pay for implementing easiness and the less complex hardware of the new proposed DCS-DBIM approach is the time penalty compared to the classical technique. However, for the ultrasound imaging, the required batch processing would last few minutes so that this time penalty for DCS-DBIM does not affect in any way its usefulness in practice.

**Table 1:** The normalized errors and runtimes of the DBIM and DCS-DBIM methods through iterations with different *N*_*t*_ and *N*_*r*_

Number of Transmitters (*N*_*t*_) and Receivers (*N*_*r*_)	Methods	Normalized error from the first iteration to the eighth iteration								Runtime (seconds)
*N*_*t*_ = *N*_*r*_ = 6	DBIM	0.8682	0.8438	NaN	NaN	NaN	NaN	NaN	NaN	69.692787
(*r* = 0.082)	DCS-DBIM	1.2077	1.2102	1.2105	1.2105	1.2105	1.2105	1.2105	1.2105	42.139530
*N*_*t*_ = *N*_*r*_ = 8	DBIM	0.7964	0.7515	0.7490	0.7489	0.7489	NaN	NaN	NaN	61.308944
(*r* = 0.145)	DCS-DBIM	1.1587	1.1718	1.1721	1.1721	1.1721	1.1721	1.1721	1.1721	47.157182
*N*_*t*_ = *N*_*r*_ = 10	DBIM	0.7305	0.6811	0.6779	0.6772	0.6771	0.6770	0.6770	0.6770	40.428921
(*r* = 0.227)	DCS-DBIM	1.1123	1.1224	1.1226	1.1226	1.1226	1.1226	1.1226	1.1226	53.017146
*N*_*t*_ = *N*_*r*_ = 12	DBIM	0.6808	0.6140	0.6083	0.6073	0.6070	0.6069	0.6069	0.6069	48.419012
(*r* = 0.327)	DCS-DBIM	0.8834	0.8945	0.8950	0.8950	0.8950	0.8950	0.8950	0.8950	63.057426
*N*_*t*_ = *N*_*r*_ = 14	DBIM	0.9367	0.6457	0.5824	0.5547	0.5398	0.5308	0.5254	0.5218	55.754608
(*r* = 0.444)	DCS-DBIM	0.7025	0.7084	0.7085	0.7085	0.7085	0.7085	0.7085	0.7085	90.422976
*N*_*t*_ = *N*_*r*_ = 16	DBIM	0.5272	0.4629	0.4585	0.4576	0.4572	0.4570	0.4570	0.4570	66.137258
(*r* = 0.581)	DCS-DBIM	0.1604	0.1078	0.1082	0.1082	0.1082	0.1082	0.1082	0.1082	185.779095
*N*_*t*_ = *N*_*r*_ = 18	DBIM	0.8196	0.5734	0.4919	0.4480	0.4176	0.3948	0.3773	0.3632	77.524221
(*r* = 0.735)	DCS-DBIM	0.1240	0.0342	0.0338	0.0337	0.0337	0.0337	0.0337	0.0337	251.473327
*N*_*t*_ = *N*_*r*_ = 20	DBIM	0.4749	0.2760	0.2356	0.2225	0.2158	0.2115	0.2086	0.2066	94.343578
(*r* = 0.907)	DCS-DBIM	0.2243	0.0689	0.0672	0.0670	0.0670	0.0669	0.0668	0.0668	237.118941
*N*_*t*_ = *N*_*r*_ = 22	DBIM	0.4604	0.2106	0.1598	0.1353	0.1209	0.1110	0.1036	0.0973	112.128716
(*r* = 1.098)	DCS-DBIM	0.3255	0.0777	0.0729	0.0724	0.0723	0.0721	0.0719	0.0718	234.584982
*N*_*t*_ = *N*_*r*_ = 24	DBIM	0.5754	0.2832	0.1321	0.0942	0.0725	0.0641	0.0534	0.0632	125.724742
(*r* = 1.306)	DCS-DBIM	0.3870	0.0310	0.0197	0.0192	0.0190	0.0188	0.0186	0.0184	225.159681
*N*_*t*_ = *N*_*r*_ = 26	DBIM	0.5545	0.1933	0.1141	0.0846	0.0685	0.0585	0.0516	0.0464	144.661175
(*r* = 1.533)	DCS-DBIM	0.1768	0.0129	0.0066	0.0064	0.0064	0.0064	0.0064	0.0064	201.250795
*N*_*t*_ = *N*_*r*_ = 28	DBIM	0.4905	0.1680	0.0858	0.0570	0.0417	0.0329	0.0271	0.0229	170.032561
(*r* = 1.778)	DCS-DBIM	0.1338	0.0080	0.0031	0.0030	0.0029	0.0029	0.0029	0.0029	238.488285
*N*_*t*_ = *N*_*r*_ = 30	DBIM	0.5971	0.3079	0.2179	0.1602	0.1215	0.0948	0.0764	0.0633	212.923908
(*r* = 2.041)	DCS-DBIM	0.1466	0.0062	0.0022	0.0022	0.0022	0.0022	0.0022	0.0022	263.288455

**Fig. 7:**
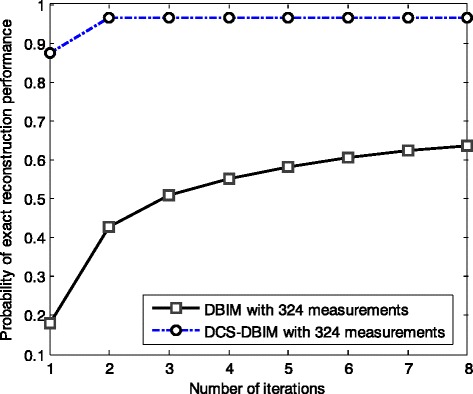
Probability of exact reconstruction performance comparison of the conventional and proposed methods

For the normalized error after *N*_*sum*_ iterations, the image reconstruction quality of the DCS-DBIM method is worse than the DBIM method when *r* <0.5 and is significantly better than the one of the conventional method when *r* >0.5. In case of *r* <0.5, although the reconstruction quality of the proposed method is not as good as the conventional method, it can successfully reconstruct the target function when *r* is very small (in case of *r* = 0.082 and 0.145). Meanwhile, the conventional method cannot reconstruct the target function (i.e. NaN in Table 1). In case of *r* >0.5, the reconstruction quality of the proposed method is significantly better than conventional method. We are concerned in practice the case that offers the best performance with a small number of measurements. Thus, we are interested in the case of *r* = 0.735 (i.e. 324 measurements) that offers a much better performance compared to the conventional method, as shown in Fig. 7.

In general, the simulation results have demonstrated that the DCS-DBIM method is a very robust tool with a very high-quality reconstruction. It is really a very promising approach in practical applications of modern biomedical imaging technology.

**Fig. 8:**
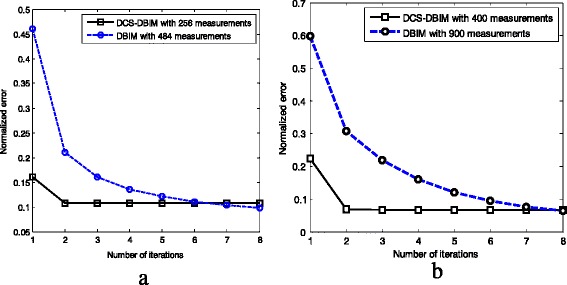
**a** Normalized error comparison of the (484 measurements) conventional and (256 measurements) proposed methods; **b** Normalized error comparison of the (900 measurements) conventional and (400 measurements) proposed methods

The error performances of both the DCS-DBIM method (in case of *N*_*t*_ = *N*_*r*_ = 16, i.e. the number of measurements = 16 × 16 = 256) and the conventional DBIM one (in case of *N*_*t*_ = *N*_*r*_ = 22, i.e. number of measurements = 22 × 22 = 484) are shown in Fig. 8a. Although the number of measurements of the DCS-DBIM method is approximately half the one of the DBIM method, these methods offer the same image reconstruction quality after the sixth iteration step. With the same normalized error, DCS-DBIM method only needs 3 iterations to complete while the DBIM method requires 6 iterations. Therefore, in this scenario, when using the proposed method, we save half of number of measurements and iterations. Furthermore, it is also shown in Fig. 8b that the proposed method still offers a very good performance (with 400 measurements), compared to the conventional method (with 900 measurements). However, as mentioned above, the runtime for the proposed method is much longer.

**Fig. 9:**
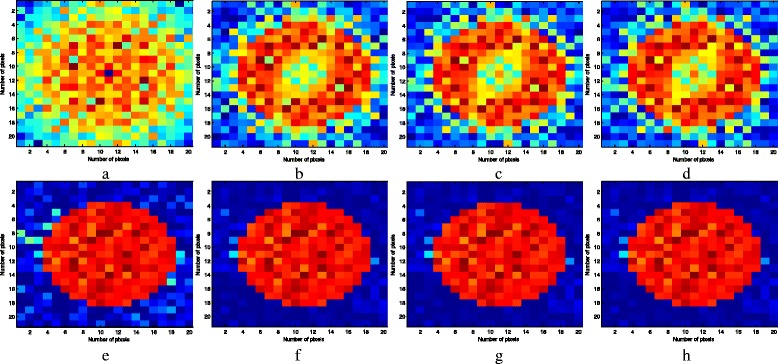
The reconstructed results of the DBIM and DCS-DBIM approaches through iterations 1 to 4 in case of *N*_*t*_ = *N*_*r*_ = 16, *r* = 0.581. Figures **a**, **b**, **c**, and **d** are of the DBIM approach and Figs. **e**, **f**, **g**, and **h** are of the DCS-DBIM approach

**Fig. 10:**
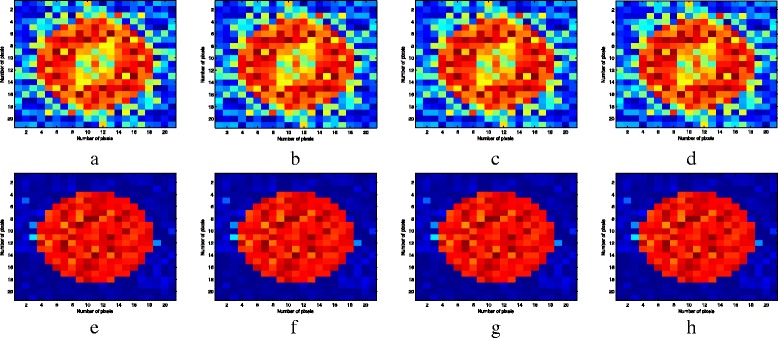
The reconstructed results of the DBIM and DCS-DBIM approaches through iterations 5 to 8 in case of *N*_*t*_ = *N*_*r*_ = 16, *r* = 0.581. Figures **a**, **b**, **c**, and **d** are of the DBIM approach and Figs. **e**, **f**, **g**, and **h** are of the DCS-DBIM approach

The reconstructed results of the DBIM and DCS-DBIM approaches through iterations 1 to 8 in case of *N*_*t*_ = *N*_*r*_ = 16 (i.e. *r* = 0.581) are depicted in Figs. 9 and 10. It can be seen that the convergence rate is obtained very fast by the DCS-DBIM approach just after the first few iterations and is not largly affected by noise. In contrast, the DBIM approach has a very low convergence rate and it is much affected by noise. With this observation, the proposed approach offers a much better quality than the conventional approach.

**Fig. 11:**
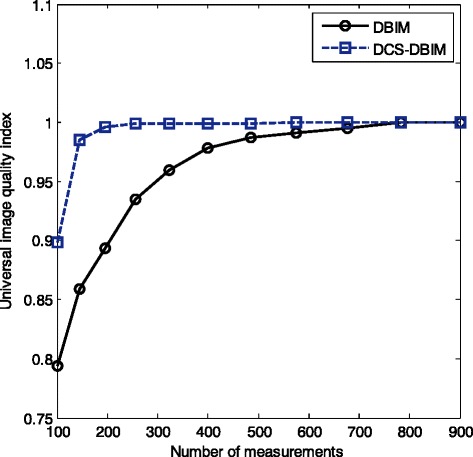
Universal image quality index comparison of the DBIM and DCS-DBIM in accordance with different number of measurements

The normalized error and similar performance indexes such as Mean Normalized Absolute Error (MNAE), Root Mean Squared Error (RMSE) are used to evaluate the reconstruction of ultrasound tomography in some relative work [9, 29–32]. In this paper, we add one more performance index (Q-index) to compare our work (DCS-DBIM) with DBIM. Universal image quality index (or Q-index) [33] comparison of the DBIM and DCS-DBIM in accordance with different number of measurements is shown in Fig. 11. The obtained results have shown that the Q-index of the DCS-DBIM method is much better in the range of small number of measurements. As shown in Fig. 11, a small number of measurements (about 200 measurements) in the DCS-DBIM method can also provide the Q-index which is equivalent to a large number of measurements (about 800 measurements) in the DBIM method. Besides, when the number of measurements is small and moderate (less than or equal to N^2^), as shown in Fig. 8, we found that the quality of the DCS-DBIM method is much better than that of the DBIM method.

**Fig. 12:**
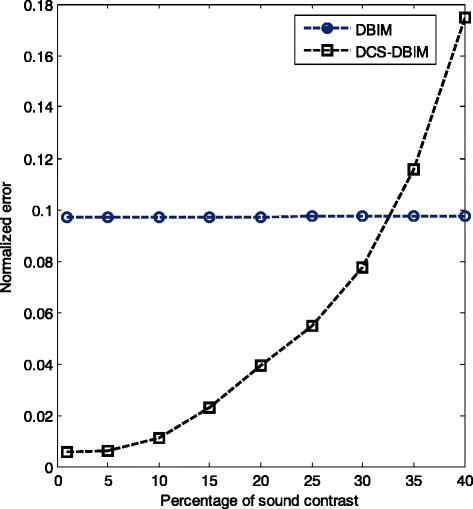
Reconstruction performance with different values of sound contrast of the DBIM and DCS-DBIM methods (N_t_ = N_r_ = 22)

Reconstruction performances with different values of the sound contrast of the DBIM and DCS-DBIM methods are shown in Fig. 12. It can be seen that the DCS-DBIM method is quite sensitive to the level of sound contrast. As shown in Fig. 12, when the sound contrast increases, the normalized error also increases. The normalized error has the smallest value when the sound contrast is about 1%. Meanwhile, the DBIM method is relatively stable when changing sound contrast (as the sound contrast increases, the normalized error increases slightly, and almost does not change much). Because the purpose of ultrasound tomography is to detect tumors in early stage, sound contrast is very small, about a few percent. Therefore, in practice, we only consider the small sound contrast. In summary, in case of small sound contrast, the DCS-DBIM method is far more effective than the DBIM method.

### Performance evaluation of the DCS-DBIM and CS-DBIM

**Fig. 13:**
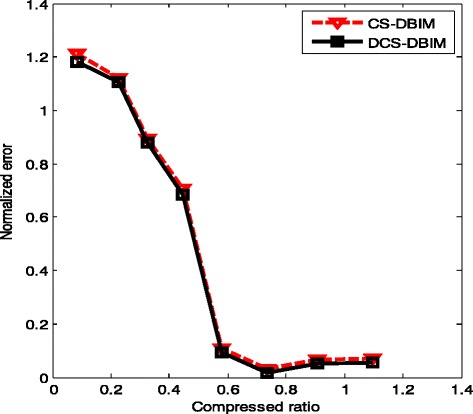
Normalized error of the CS-DBIM and DCS-DBIM methods with different compressed ratios

The performances of DCS-DBIM and DBIM have been discussed and compared in the last part. We are going to compare DCS-DBIM and CS-DBIM performances. In Fig. 13, we have seen that the DCS-DBIM offers a performance almost similar to that of the CS-DBIM.

The numerical simulation results are quite consistent with previous work on deterministic compressed sampling technique [10]. Furthermore, because the implementation of compressed sampling technique is based on random sampling, this means that transducers are randomly distributed on measurement system. This random structure leads to a complex hardware implementation. If we apply the deterministic compressed sampling technique which uses a non-linear deterministic system that apparently acts like a random system [21], its hardware implementation of the deterministic structure is simpler than the random one. Note that the exact reconstruction of the deterministic compressed sampling technique is guaranteed like the random one.

**Fig. 14:**
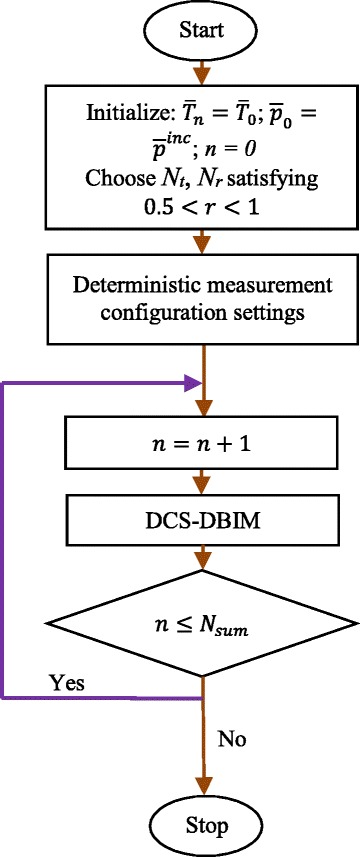
Proposed flowchart of the DCS-DBIM procedure

The implementation procedure of the DCS-DBIM is presented in Fig. 14. Assume that number of pixels of the ideal object function (*N*) and total number of iterations (*N*_*iter*_) are given. The flowchart presented in Fig. 14 starts with the initialization of three parameters ${\overline{O}}_n,{\overline{p}}_0$, and *n* (${\overline{O}}_n={\overline{O}}_0;{\overline{p}}_0={\overline{p}}^{inc}; n= 0$). In addition, *N*_*t*_ *N*_*r*_ is also chosen so that it is smaller than *N* ^2^, pixels of the ideal object function (for the best case, 0.5 < *r* <1). We set up the deterministic measurement configuration based on deterministic filter. Next, the DCS-DBIM algorithm is used to reconstruct the target in *N*_*sum*_ iterations. The output result of the procedure is the reconstructed function after *N*_*iter*_ iterations.

## Discussion

Based on inverse scattering theory, the DBIM is a well-known quantitative imaging approach for detecting very small targets thanks to its mechanical properties. Deterministic compressive sampling technique is a promising approach for feasible hardware implementation in practical applications. This paper has successfully applied DCS technique for setting up the measurement configuration for the DBIM, and then the target is reconstructed using *l*_1_ least square problem in order to improve the quality of the image reconstruction. This method also offers a simpler setting compared to the others. Simulation scenarios of sound contrast reconstruction were implemented to demonstrate the very good performance of this method. These results have shown that the gain of this new approach merits practical considerations. For practical purposes, the reconstruction of three-dimensional (3D) image is done by using many 2D slices at different positions of z-axis; indivual processing outcomes are finally all merged together [34]. Therefore, the core issue is that we need to acquire good 2D slices.

With current transducer array technology, one transducer can both transmit and receive ultrasound signal. Thus, when setting up the actual measurement configuration, depending on imaging quality requirement, we can arrange transducers on the measurement system so that the distance between two transducers can be 1°, 2°, etc.… If the distance between two transducers is small, we can arrange multiple transmitters and receivers on the measurement system such that we can reconstruct high-resolution images (i.e.large number of pixels in the range of interest); reciprocally, if the distance between two transducers is large, the number of transmitters and receivers will be less. Therefore, we can reconstruct low-resolution images. The number of transmitters and receiverswill have to be chosen in the acceptable range in order to reconstruct good-enough image, i.e. 0.5 < *r* < 1. However, to be more reasonable, we should arrange the configuration such that the distance between two transducers are small, about 1°. With this arrangement, when we createthe deterministic sequence of the DCS, the indexes of this sequence correspond to the positions of transducers on the measurement system. This creates a random-like system, and thus ensures conditions of reconstruction in compressed sampling technique [6, 7]. This set-up does not make the imaging process more complex. In fact, not all transducers in the measurement system work, only transducers whose indexes coincide with the ones of the deterministic sequence. Therefore, the volume of calculation only depends on the number of active transducers on the measurement system.

## Conclusion

Inspired of easier hardware implementation of deterministic CS, in this paper, we have proposed the deterministic measurements in the detection geometry configuration and the image reconstruction process has been implemented using *l*_1_ regularization. The simulation results of the proposed method have demonstrated the high performance of the proposed approach, where the normalized error is approximately 90% reduced, compared to the conventional DBIM approach. With the same quality, we can save half of number of measurements and only use two iterations when using the proposed method. Furthermore, numerical simulation results also indicate that CS and DCS techniques offer equivalent image reconstruction quality. However, DCS has the advantage of being able to execute much more convenient hardware. With current transducer technology and using the slicing technique that transforms 3D images to several 2D images, we can produce multiple sliced images at the same time. This will significantly reduce imaging time for patients and make ultrasound tomography imaging a real-time imaging tool. To implement this approach in practice while keeping the cost at an acceptable level, the following two possibilities might be considered: 1) We only need a 2D measurement system; when imaging different slices, the measurement system will shift along the z axis to produce images of different slices. However, this will take more time and the shifted measurement system will cause mechanical errors; 2) We set up the measurement system following women’s breast shape; the process of imaging at different slices will simultaneously take place. However, can we arrange transducers on various slices differently and sparsely? If this can be done, set-up costs would significantly reduce. This is a fascinating issue for further study.

## Abbreviations

BIM: Born iterative method
CS: Compressed sensing
CT: Computed tomography
DBIM: Distorted born iterative method
DCS: Deterministic compressed sensing
DCS-DBIM: Deterministic compressed sensing- distorted born iterative method
EPR: Electron paramagnetic resonance
HRNG: Hardware random number generator
MRI: Magnetic resonance imaging
PRNG: Pseudo-random number generator
RIP: Restricted Isometry property
UT: Ultrasound tomography
